# Pancreatogenic Diabetes in Children With Recurrent Acute and Chronic Pancreatitis: Risks, Screening, and Treatment (Mini-Review)

**DOI:** 10.3389/fped.2022.884668

**Published:** 2022-04-26

**Authors:** Melena D. Bellin

**Affiliations:** University of Minnesota Medical School and Masonic Children’s Hospital, Minneapolis, MN, United States

**Keywords:** DM, T3cD, islet, endocrine, exocrine, pancreatic, insulin

## Abstract

Up to 9% of children with acute recurrent pancreatitis (ARP) or chronic pancreatitis have pancreatogenic diabetes mellitus (DM), and this risk likely increases as they age into adulthood. Risk factors for pancreatogenic DM in children vary depending on the clinical cohort but may include pancreatic atrophy, exocrine insufficiency, pancreatic calcifications, obesity/metabolic syndrome features, or autoimmune diseases. Knowledge regarding disease pathology is extrapolated nearly entirely from studies in adults. Insulin deficiency is the primary defect, resulting from islet loss associated with pancreatic fibrosis and cytokine-mediated β-cell dysfunction. Beta cell autoimmunity (type 1 diabetes) should also be considered as markers for this have been identified in a small subset of children with pancreatogenic DM. Hepatic insulin resistance, a deficient pancreatic polypeptide state, and dysfunctional incretin hormone response to a meal are all potential contributors in adults with pancreatogenic DM but their significance in pediatrics is yet unknown. Current guidelines recommend yearly screening for diabetes with fasting glucose and hemoglobin A1c (HbA1c). Insulin in the first-line pharmacologic therapy for treatment of pancreatogenic DM in children. Involvement of a multidisciplinary team including a pediatric endocrinologist, gastroenterologist, and dietitian are important, and nutritional health and exocrine insufficiency must also be addressed for optimal DM management.

## Introduction

While rare in childhood, acute recurrent pancreatitis (ARP) and chronic pancreatitis (CP) are associated with high disease morbidity. Affected children most often present with severe episodic or persistent abdominal pain (1, 2). As the disease progresses, however, these children are at risk for pancreatic dysfunction, namely pancreatic exocrine insufficiency and pancreatogenic diabetes mellitus (DM) (3). Pancreatogenic DM is broadly defined as diabetes resulting from a primary exocrine disease of the pancreas, and includes diabetes that occurs secondary to acute or chronic pancreatitis. There are various synonyms used for this condition in the literature, including type 3c diabetes, pancreatogenous diabetes, or pancreatic diabetes (4). This mini-review will highlight the current knowledge on the potential mechanisms driving pancreatogenic DM, and screening and treatment guidelines for children with ARP and CP who may be at risk (Table 1).

**TABLE 1:** Summary of the potential risk factors for and pathophysiology of pancreatogenic DM in children with ARP and CP, and screening and treatment recommendations, limited by a small number of studies in children to date.

Potential risk factors	Pathophysiology (suspected)	Screening	Treatment
• Exocrine insufficiency	• Insulin deficiency ± ? (unclear role of)	• Yearly HbA1c and fasting glucose	• Insulin
• Pancreatic atrophy	• *Insulin resistance*	• OGTT as indicated	• Multi-disciplinary team
• Obesity/metabolic syndrome	• *PP deficiency*	• MMTT (research or serial assessments)	• Nutritional management including PERT if indicated
• Pancreatic calcifications	• *Incretin dysfunction*		
• Autoimmune diseases	• *β-cell autoimmunity*		

## Prevalence of Pancreatogenic Diabetes in Children

About 4–9% of children with ARP and CP have pancreatogenic diabetes (1, 3, 5–7). This is much higher than the prevalence of diabetes in the general pediatric population with only around 0.25% of children having any form of diabetes (8). However, considering the rarity of pediatric ARP and CP, probably affecting no more than ∼0.1% of children, pediatric pancreatogenic DM is rare (9). In contrast, in adults with CP, 25–80% have diabetes, with duration of disease, pancreatic surgical history, pancreatic calcifications, and comorbid exocrine insufficiency all impacting risk (4, 10–12). This difference in prevalence between children and adults likely represents the natural progression of pancreatitis, in that the pancreatic parenchyma becomes more damaged with a longer duration of disease, as well as the higher risk in general for diabetes with aging. In hereditary forms of CP, DM prevalence increases steadily with age; available natural history studies suggest at least 60–70% of those with hereditary pancreatitis will develop pancreatogenic diabetes in their lifetime, with a median age of onset around 50 years (13). In addition, adults are at higher risk for type 2 DM and may have an overlap of type 2 DM and pancreatogenic DM (8). Thus, in evaluating children with pancreatitis one must consider the current and future risk for pancreatogenic DM.

In children, pancreatogenic DM risk may be elevated when pancreatic exocrine insufficiency, obesity, or pancreatic atrophy are also present, and at an older age (3, 5, 6). Pancreatic calcifications may also be a risk factor but this has not been consistently shown in children, probably due to the low frequency of advanced calcific disease compared to the adult population (3). Total pancreatectomy with islet autotransplantation (TPIAT) is gaining traction in the U.S. as a treatment for severe forms of hereditary pancreatitis. Because of the resection of the pancreas and islet loss, TPIAT is associated with a high rate of post-surgical insulin dependent diabetes (14). This select group of children undergoing TPIAT for intractable ARP or CP has been specifically excluded when calculating the 4–9% prevalence of pancreatogenic DM in children.

## Mechanisms of Pancreatogenic Diabetes (Studies From Adults)

### Insulin Secretion

Current research into the pathophysiology underling pancreatogenic DM has been limited largely to research enrolling adult participants or using adult tissue samples. The primary defect is inadequate insulin secretion from both irreversible islet loss and beta cell dysfunction. Histopathology studies of pancreas tissue in adults with CP demonstrate reduced beta cell area in CP with DM (15). Likewise, stimulatory tests of insulin secretion show reduced insulin secretion compared to healthy controls, and a correlation between reduced islet and reduced exocrine function, supporting the concept that insulin secretory defects occur because of scarring through the exocrine pancreas that incidentally damages the islet tissue (16, 17). Reduced first phase and post-meal insulin secretion is apparent even prior to frank onset of pancreatogenic DM (18). Late in the course of disease, after insulin deficient pancreatogenic DM develops, glucagon secretion from α cells is also reduced, again attributed to an overall islet loss (19). This lack of glucagon production is important clinically, since it may predispose patients to a higher risk for hypoglycemia.

More recently, it has been suggested that beta cell function may be impaired from intrapancreatic inflammation even prior to islet loss. Tissues resected from surgical patients with CP with or without pancreatogenic DM showed elevated pancreatic cytokines in both groups including greatly elevated IL-1β, IL-6, IL-8, TNFα, IL-10, and INFγ compared to healthy control pancreas tissue. Notably, INFγ was significantly higher in the CP- pancreatogenic DM group versus CP without diabetes (20). These observations have led to the hypothesis that β-cell dysfunction may occur from inflammation even before frank loss of islet tissue.

**FIGURE 1:**
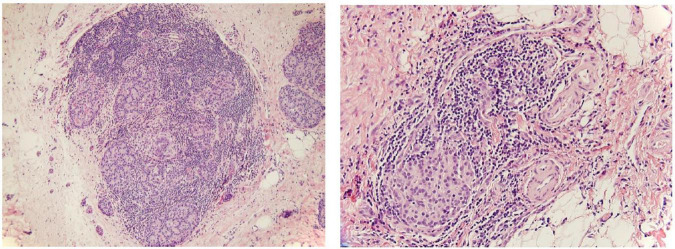
Example of the insulitis characteristic of type 1 diabetes in a patient with chronic pancreatitis. Lymphocytic inflammation surrounds and infiltrates the islet tissue while the exocrine tissue is largely fibrotic from CP.

The classic paradigm has been that insulin deficiency results from non-specific pancreatic damage and inflammation, that is the islets are simply an “innocent bystander” that are not directly targeted. However, in the INSPPIRE study, around one quarter of the children with pancreatogenic DM also were reported to have islet autoantibodies and pancreatogenic DM was higher in children with other autoimmunity, raising some speculation for a sub-population of children who develop type 1 DM—an autoimmune attack directed at the β-cells—in the setting of CP or ARP (3). β-cell autoantibodies and insulitis have been incidentally reported in some children with CP undergoing TPIAT (Figure 1), and adults in the North American Pancreatitis-2 (NAPS2) study with CP or ARP and pancreatogenic DM had a 10% risk of islet autoantibody positivity, even after excluding insulin autoantibodies (which can be falsely positive with insulin treatment) from the analysis (21, 22). Thus, whether a small portion of children may have autoimmune islet loss in pancreatogenic DM needs additional investigation.

### Insulin Sensitivity

Beta cell function is comprised of both insulin secretion and insulin sensitivity. Individuals who are insulin resistant must secrete more insulin to overcome the impaired insulin responsiveness, and if unable to do so, DM may result (23). In adults with CP in the NAPS-2 cohort, in addition to canonical pancreatitis risk factors, obesity and a family history of DM—classic type 2 DM risk factors—increased the risk for pancreatogenic DM (11). Genetic risk scores for diabetes in pancreatogenic DM also resemble that seen in type 2 diabetes (24). There are conflicting data on measures of peripheral insulin sensitivity, but data from euglycemic clamp techniques differentiating peripheral and hepatic insulin sensitivity suggest specific hepatic insulin resistance may contribute to diabetes pathology, at least in adult studies mostly comprised of men with alcohol-mediated pancreatitis (25, 26). Visceral adiposity has also been shown to be higher in adults with CP who have pancreatogenic DM when compared to their non-diabetic counterparts (27).

Insulin resistance and metabolic syndrome risks have not been directly studied in children, but the children with pancreatogenic DM in INSPPIRE were more likely to also have hypertriglyceridemia (3).

### Other Hormones Including Incretins and Pancreatic Polypeptide

Available data from small cross-sectional studies suggest that pancreatic polypeptide (PP) produced and secreted by the PP-cells of the islets is deficient in pancreatogenic DM and may serve as a biomarker in differentiating pancreatogenic DM from type 2 DM (28–30). It has also been proposed that the PP deficiency itself may contribute to the hepatic insulin resistance, as infusion of PP in one small series improved hepatic insulin sensitivity (25). However, the role of PP requires further ongoing study, especially in children.

The incretin hormones glucagon like peptide-1 (GLP-1) and glucose-dependent insulinotropic polypeptide (GIP) are released by the enteroendocrine cells of the gut and play an important role in glucose homeostasis by augmenting insulin secretion in response to oral nutrient ingestion. GLP-1 and GIP are reduced in CP, presumably due to exocrine insufficiency and malabsorption impairing the usual enteroendocrine response (31, 32). Administration of pancreatic enzyme replacement therapy can improve GLP-1 and GIP levels and increase insulin secretion in CP, but whether this results in improved glycemia is unclear (32). To date the role of PP and incretins as biomarkers or drivers of pancreatogenic DM in children have not been studied.

## Screening for Diabetes in Children With a History of Pancreatitis

Children diagnosed with ARP and CP should be screened yearly for diabetes given the known high risk in this population. This can be done easily in the clinic with fasting glucose and HbA1c on a yearly basis (33, 34). In cases of pre-DM or high suspicion for pancreatogenic DM, a 2 h oral glucose tolerance test (OGTT) may be considered to detect early DM with better sensitivity. Mixed meal tolerance tests (MMTT) may also be obtained as a more physiologic measure of islet function and glycemia, and are particularly useful in the research setting or for trends over time. However, MMTT lacks the diagnostic cut-offs for pre-DM and DM that have been established for OGTT so are less desirable as a screening tool for DM (34).

The diagnosis of pancreatogenic DM should be made according to standard American Diabetes Association criteria. By ADA criteria, diabetes is diagnosed when two tests on the same or separate mornings are in the abnormal DM range: HbA1c ≥ 6.5%, fasting glucose ≥ 126 mg/dL, or 2 h OGTT glucose ≥ 200 mg/dL. A random glucose ≥ 200 mg/dL with classic symptoms of DM is also considered diagnostic (35). Islet autoantibody panels (including insulin autoantibodies, glutamic acid decarboxylase, IA-2, or zinc transporter-8 antibodies) should be considered, especially with symptomatic blood glucose ≥ 200 mg/dL. Pre-DM is diagnosed by HbA1c 5.7–6.49% or fasting glucose 100–125 mg/dL, or 2 h OGTT glucose 140–199 mg/dL (35). The risk for progressing from pre-DM to pancreatogenic DM in children with CP or ARP is unknown.

## Treatment and Special Considerations

First-line pharmacologic treatment of pancreatogenic DM in children is typically with insulin therapy. In the INSPPIRE cohort, insulin was the most common treatment (used in 20/24 cases) (3). Insulin addresses the main physiologic defect which is insulin deficiency, and may be administered by subcutaneous injections of basal and/or bolus insulin therapy, or by continuous subcutaneous infusion using an insulin pump. While traditional glucometers remain an acceptable option for blood glucose monitoring, continuous glucose monitors are now preferred for better monitoring and safety in children treated with rapid-acting insulin (35).

Metformin may be considered when features of insulin resistance are present, including obesity, acanthosis nigricans or a strong family history of T2DM (36). Although GLP-1 agonists are now approved for pediatric use in type 2 DM and obesity, this class of medications has not been studied for pancreatogenic DM in adults or children. There is a possible, but controversial, increased risk of pancreatitis (and pancreatic cancer) with GLP-1 agonists which raise a serious safety concern for their use in children who already have ARP or CP (37). Aside from the debated pancreatitis risk, nausea and delayed gastric emptying are common and well-known side effects and thus the tolerability of this class of medications would likely be poor in children with pancreatogenic DM (37). For these reasons, current care guidelines for children and adults with pancreatogenic DM have recommended against their use. Other non-insulin medications are not approved by the FDA for use in children.

A pediatric endocrinologist, diabetes educator, pediatric gastroenterologist, and pediatric dietitian should all be part of the care team for children with pancreatogenic DM. Maintaining a normal body weight, avoiding both underweight and overweight status, should be a target of nutritional management. Co-morbid exocrine insufficiency or malabsorption must be appropriately addressed with pancreatic enzyme replacement therapy. Adherence and adequacy of dosing should be assessed at clinic visits. Because insulin therapy is often dosed based on meal intake (an insulin to carbohydrate ratio), malabsorption could lead to glycemic variability, with inconsistent insulin responses to meal dosed insulin and more hyper- or hypoglycemia episodes (38).

## Discussion, Conclusion, and Future Directions

In summary, children with ARP and CP should be considered high risk for developing DM. Pancreatogenic DM has an overall prevalence of 4–9% in children with ARP and CP, although the risk will be even higher after pancreatectomy procedures including TPIAT. Pancreatogenic DM results primarily from insulin deficiency, as islets are lost from progressive fibrotic damage to the pancreas and/or are impaired by intrapancreatic inflammation, with a speculative role for directed β cell autoimmunity in a small subset. However, there may be an important contributing role of insulin resistance or dysfunction of associated hormones including PP, GLP1, or GIP. Importantly, nearly all mechanistic studies have been performed to date in adult patients, and thus our direct data regarding pathophysiology of disease in children are limited. Prospectively designed studies are needed in children with ARP and CP.

Children with ARP and CP need to be screened annually for DM and this is easily done by fasting glucose and HbA1c levels. Potential type 1 or type 2 DM should still be considered when assessing the child with pancreatitis who presents with new onset DM. First-line treatment is typically with insulin therapy to address the primary physiologic defect, but clinical trials of diabetes treatment in this unique group are lacking. Other CP factors including pancreatic exocrine insufficiency and nutritional limitations must be considered and addressed by a multi-disciplinary team as part of the management of the child with pancreatogenic DM.

## Author Contributions

MB drafted the manuscript.

## Conflict of Interest

The author declares that the research was conducted in the absence of any commercial or financial relationships that could be construed as a potential conflict of interest. The handling editor VM declared a shared consortium, INSPPIRE consortium (International Study Group for Pediatric Pancreatitis - In Search for a Cure), with the author MB at the time of review.

## Publisher’s Note

All claims expressed in this article are solely those of the authors and do not necessarily represent those of their affiliated organizations, or those of the publisher, the editors and the reviewers. Any product that may be evaluated in this article, or claim that may be made by its manufacturer, is not guaranteed or endorsed by the publisher.
